# Which sequence should be used in the thorax magnetic resonance imaging of COVID-19: a comparative study

**DOI:** 10.55730/1300-0144.5687

**Published:** 2023-08-11

**Authors:** Ayşe Şule ATEŞ, Burak YAĞDIRAN, Onur TAYDAŞ, Ömer Faruk ATEŞ

**Affiliations:** 1 Department of Chest Diseases, Faculty of Medicine, Sakarya University, Sakarya, Turkiye; 2 Department of Radiology, Faculty of Medicine, Başkent University, Ankara, Turkiye; 3 Department of Radiology, Faculty of Medicine, Sakarya University, Sakarya, Turkiye

**Keywords:** COVID-19, magnetic resonance imaging, sequence, interobserver intraclass coefficients, interobserver agreement

## Abstract

**Background and aim:**

To evaluate and compare magnetic resonance imaging (MRI) sequences that could potentially be used in the diagnosis of coronavirus disease 2019 (COVID-19).

**Materials and methods:**

Included in the study were 42 patient who underwent thorax computed tomography (CT) for COVID-19 pneumonia and thorax MRI for any reason within 24 h after CT. The T2-weighted fast spin echo periodically rotated overlapping parallel lines with enhanced reconstruction (PROPELLER) (T2W-FSE-P), fast imaging employing steady-state acquisition, T2 fat-saturated FSE, axial T1 liver acquisition with volume acceleration (LAVA) and single-shot FSE images were compared in terms of their ability to show COVID-19 findings.

**Results:**

The mean age of the patients was 47.2 ± 24 years. Of the patients, 22 were male (52.4%) and 20 (47.6%) were female. The interobserver intraclass coefficient (ICC) for the image quality score was the highest in the T2W-FSE-P sequence and lowest in the T1 LAVA sequence. All of the lesion-based evaluations of the interobserver agreement were statistically significant, with the kappa value varying between 0.798 and 0.998.

**Conclusion:**

All 5 sequences evaluated in the study were successful in showing the parenchymal findings of COVID-19. Since the T2W-FSE-P sequence had the best scores in both interobserver agreement and ICC for the image quality score, it was considered that it can be included in thorax MRI examinations to assist the diagnosis of COVID-19.

## 1. Introduction

In late December 2019, severe acute respiratory syndrome caused by a new type of coronavirus, called coronavirus disease 2019 (COVID-19), was defined for the first time in China [1]. The disease spread rapidly in China, and then across the world, soon being declared a pandemic by the World Health Organization [2]. This new type of coronavirus enters cells through angiotensin-converting enzyme 2 receptors [3]. Therefore, it causes interstitial involvement before parenchymal involvement in the lungs [4]. Although the most common clinical symptoms are cough and fever, some patients may also have nonspecific symptoms, such as weakness, headache, and dyspnea. Since the disease can rapidly progress to severe pneumonia, a rapid diagnosis is required [5]. As a diagnostic test, the gold standard method is the real-time reverse transcription-polymerase chain reaction (RT-PCR) test. However, RT-PCR may be insufficient due to various reasons, such as false-negative results and the unavailability of PCR kits in some regions.

Although not recommended routinely, chest computed tomography (CT), whose sensitivity reaches 97% in some studies, has a very important place in the diagnostic evaluation of suspected COVID-19 cases, especially in the high-risk patient group with comorbidities, those who do not respond to treatment, and those whose clinical state deteriorates during follow-up. In these patients, chest CT is very important for the follow-up of the disease course and the early diagnosis of complications that may occur. Typical CT findings are diffuse or lower zone ground-glass opacities (GGOs), consolidation with GGOs, crazy paving pattern, and consolidation. These findings are usually located in the peripheral and posterior regions [6–8].

Despite being useful in diagnosis, CT involves ionizing radiation. In the last consensus statement of the Fleischner Association, the ionizing radiation exposure effect of CT in patients has been defined as one of its major disadvantages [9]. Even if low-dose CT protocols have been developed and are now being used, the dose-dependent and dose-independent effects of ionizing radiation applied to a very large population will be observed in the coming years [3, 10]. Therefore, as an alternative to CT, magnetic resonance imaging (MRI) appears to be the primary cross-sectional imaging method [3, 11].

The aim of this study was to evaluate and compare MRI sequences that could potentially be used in the diagnosis of COVID-19.

## 2. Materials and methods

### 2.1. Patient selection

For this retrospective study, approval was obtained from the ethics committee of our institution. Of the 361,820 patients diagnosed with COVID-19 pneumonia (positivity in at least 1 RT-PCR test + clinical confirmation) in our institution between May 2020 and September 2021, 42 of those who underwent thorax CT for COVID-19 pneumonia and thorax MRI for any reason within 24 h after CT were included in the study. MRI images were generally obtained for the following reasons: pulmonary MRA obtained for suspicion of pulmonary thromboembolism in some patients with increased D-dimer without renal failure but with limited renal function and patients with suspected cardiac involvement and therefore imaged with cardiac MRI.

The clinical and radiological findings are summarized in Table 1.

### 2.2. MRI examinations

MRI was performed with a 1.5-T system (Signa Voyager; GE Healthcare, Milwaukee, WI, USA) using a phased array body coil. The following sequences were obtained: T2-weighted fast spin echo periodically rotated overlapping parallel lines with enhanced reconstruction (PROPELLER) (T2W-FSE-P) axial and coronal, axial fast imaging employing steady-state acquisition (FIESTA), axial T2 fat-saturated FSE (T2 FSE), axial T1 liver acquisition with volume acceleration (T1 LAVA), and axial and coronal single-shot FSE (SSFSE). The MRI sequence parameters are summarized in Table 2.

### 2.3. CT examinations

CT images were obtained with a 64-row multidetector scanner (Aquilion64; Toshiba Medical Systems, Otawara, Tochigi, Japan) in 27 patients and with a 16-row multidetector scanner (Alexion; Toshiba Medical Systems) in 14 patients. The imaging parameters were as follows: slice thickness, 5 mm; matrix, 512 × 512; and automatically modulated mA, 120 kV. All of the scans were performed during inspiration and with the patients placed in the supine position.

### 2.4. Image analysis

The MRI and CT images of all of the patients were evaluated for the presence of opacity and unilateral or bilateral involvement. The number of lobes affected (n = 1–5) and number of lobes containing GGOs, consolidation and the crazy paving pattern were determined. On the CT and MRI, a density/intensity increase in which vascular boundaries could be distinguished was accepted as GGOs, while a density/intensity increase in vascular structures that could not be differentiated was considered as consolidation.

The MRI images were assessed for quality: 5, excellent no artifacts; 4, good (few artifacts); 3, moderate (of diagnostic value but impaired by artifacts); 2, poor (of no diagnostic value); and 1, not tolerated (examination could not be completed). The causes of impaired quality were attributed to ghosting, motion or patient movement artifacts, or a combination thereof. The evaluation of the images was independently undertaken by 2 radiologists (both board-certified and with 8 years of experience) with the prediagnosis of COVID-19-related pneumonia. A period of 2 weeks was allowed to pass for the evaluation of MRI images after the CT examination to prevent memory bias. If the initial opinions of the radiologists differed, a consensus was reached by examining the images together.

### 2.5. Statistical analysis

MedCalc (ver. 12, Ostend, Belgium) was used for the statistical analysis. The descriptive statistics were given as the median (minimum–maximum) and mean ± standard deviation values. Categorical variables were expressed as frequencies and percentages. The chi squared test was used for comparison of the categorical variables. The independent samples t test was used for comparison of the continuous variables with a normal distribution and the Mann-Whitney U and Kruskal Wallis tests were used for data that did not conform to the normal distribution according to the Kolmogorov-Smirnov test. In order to determine the interobserver and intermethod agreement, the Kendall coefficient of concordance (Kendall’s W) and intraclass coefficients (ICCs) were calculated. The assessment of the interobserver and intermethod agreement was evaluated using the Kendall W and Cohen Kappa coefficients. Based on the 95% confidence interval (CI) of the intraclass coefficients (ICC) estimate, values less than 0.20, between 0.20 and 0.40, between 0.40 and 0.60, between 0.60 and 0.80, and greater than 0.80 were indicative of poor, fair, moderate, substantial, and excellent reliability, respectively.

## 3. Results

A total of 42 patients were included in the study. The mean age of the patients was 47.2 ± 24 years, 22 patients were male (52.4%) and 20 (47.6%) were female. In 3 patients (7.1%), there were no COVID-19 findings on CT and MRI, while the remaining 39 (92.8%) patients had radiological findings of COVID-19. The median image quality score was 5 for the FIESTA (range: 3–5) and 4 for all of the remaining sequences (range: 2–5).

**Figure 1:**
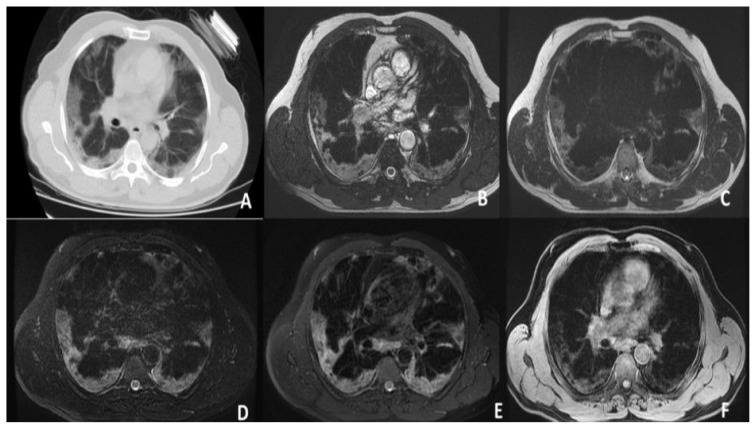
Bilaterally peripherally located GG and consolidation areas of the patient. **A**. CT. **B**. FIESTA sequence. **C**. SSFSE sequence. **D**. T2 FS sequence. **E**. T2 PROPELLER sequence. **F**. T1 LAVA-F sequence.

**Figure 2:**
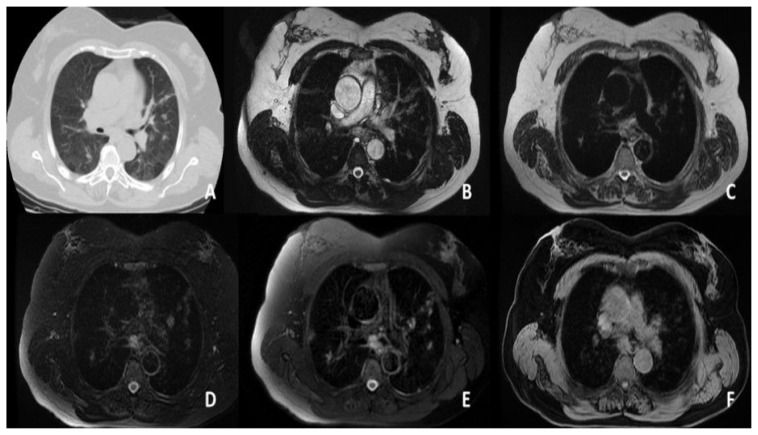
Patches of the GG and consolidation in both lungs, with more on the left. **A**. CT. **B**. FIESTA sequence. **C**. SSFSE sequence. **D**. T2 FS sequence. **E**. T2 PROPELLER sequence. **F**. T1 LAVA-F sequence.

**Figure 3:**
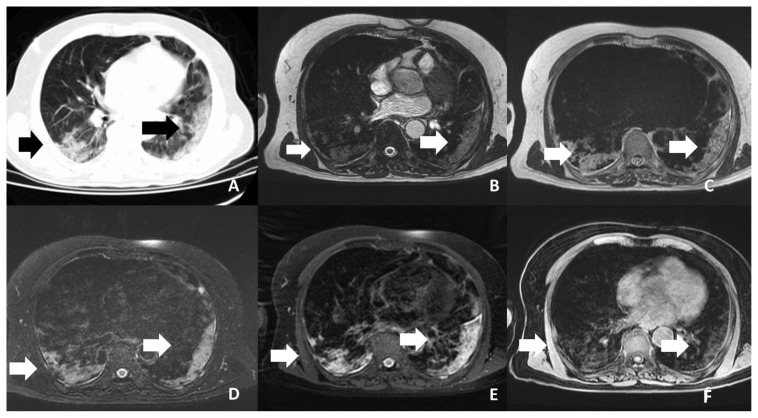
Shows bilaterally peripherally located ground glass and consolidation areas of the patient. **A**-CT **B**-FIESTA sequence **C**-SSFSE sequence **D**-T2 FS sequence **E**-T2 PROPELLER sequence **F**-T1 LAVA-F sequence

**Figure 4:**
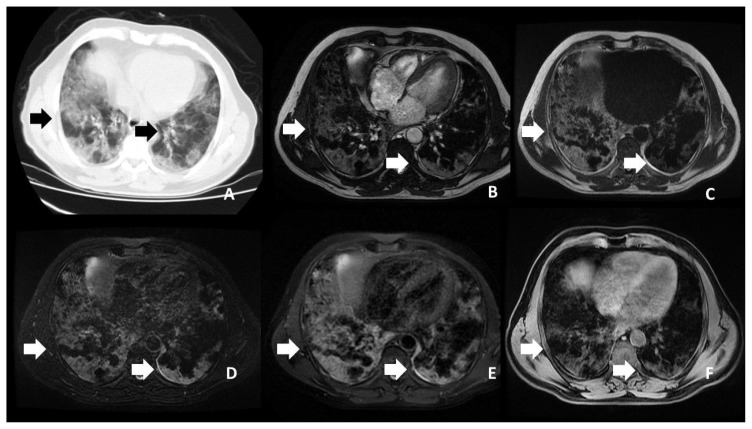
Bilaterally peripherally located crazy paving areas of the patient. **A**. CT.l **B**. FIESTA sequence. **C**. SSFSE sequence. **D**. T2 FS sequence. **E**. T2 PROPELLER sequence. **F**. T1 LAVA-F sequence.

According to the CT findings, 1 lobe was affected in 14 (35.9%) patients, 2 lobes in 10 (25.6%), 3 lobes in 7 (17.9%), 4 lobes in 5 (12.8%), and 5 lobes in 4 (10.3%). Moreover, 31 patients (79.5%) had bilateral involvement and 35 (89.7%) had multifocal involvement. There were both GGOs and consolidation in 25 patients (64.1%) (Figures 1a–1f, 2a–2f, and 3a–3f), only GGOs in 8 (20.4%) patients, and only consolidation in 6 (16.5%) patients. Air bronchogram was observed in 15 patients (38.4%), crazy paving pattern in 4 (10.2%) patients (Figures 4a–4f), and pleural effusion in 4 (10.2%) patients.

Table 3 shows the ICC values for the image quality score. The ICC was the highest in the T2W-FSE-P sequence and lowest in the T1 LAVA sequence. Table 4 shows the evaluation of the interobserver agreement in terms of radiological findings of COVID-19 between the MRI sequences. The highest ICC value belonged to the T2W-FSE-P sequence and the lowest ICC value was obtained from the T1 LAVA for all of the COVID-19 radiological findings.

The Kendall W coefficient was used to measure the interobserver agreement of the lesion-based assessment of the radiological findings (Table 5). All of the lesion-based evaluations were statistically significant, with the kappa value varying between 0.798 and 0.998. Only for the T1 LAVA sequence, was the kappa value of GGOs with consolidation and crazy paving pattern below 0.8. For all of the remaining lesions, the intermethod agreement was evaluated as excellent for all of the sequences.

## Discussion

The most important result of this study was that in the diagnosis of the parenchymal findings seen in COVID-19, all 5 MRI sequences showed high interobserver agreement and ICC for the image quality score. Among the sequences evaluated, the best performance belonged to the T2W-FSE-P. Zhao et al. compared the oxygen enhanced respiratory-gated 3-dimensional (3D) ultrashort echo time (UTE MRI) sequence with the CT images of 49 COVID-19 RT-PCR-positive patients. They reported no significant difference between CT and MRI [12], which is in agreement with the results of the present study. In another recent study, T2 TSE, T2 SSFSE, and respiratory-gated 3D radial UTE-MRI sequences were used in 23 patients with COVID-19 pneumonia, and MRI and CT were determined to have similar image quality in the examined pulmonary pathologies. No statistically significant difference was found between the 2 modalities in relation to any of pathologies [13]. In another study, high compatibility was detected between UTE-MRI and CT in the demonstration of pulmonary pathologies [14].

The chest CT examination is most commonly used in the thoracic imaging of COVID-19 pneumonia. Bilateral, peripheral, subpleural and posterior-weighted involvement is frequently detected on chest CT [15–17]. The most common finding in chest CT is GGOs, followed by reticular opacities with interlobular and intralobular septal thickening showing interstitial involvement [18, 19]. Another finding is consolidation, which can be isolated or accompanied by GGOs [20]. Consolidations accompanied by GGOs indicate the development of organized pneumonia, and there are hyaline membranes and pulmonary oedema in its physiopathology [21]. Another important finding is the crazy paving pattern, in which GGOs are seen together with superimposed interlobular and intralobular septal thickening, although it is less common than the other findings [17–22]. Other findings that are less frequent and accepted as supportive findings include pleural thickening, intralobular septal thickening, pulmonary vascular enlargement, subpleural lines, air bronchogram, and the reverse halo sign [18]. Mediastinal lymphadenopathy and pleural effusion are considered to be atypical findings, suggesting superposed bacterial infection in RT-PCR-positive patients [23].

In a recent study by Ates at al., the number and localization of consolidations and infiltrations, and the presence of pleural effusion were compared between the T2W-FSE-P sequence and CT in 32 patients. In addition, the visibility of the nodules detected on chest CT was investigated using MRI, which had not been previously examined in other studies in the literature. According to the results, there was no statistically significant difference between the chest CT and MRI findings of infiltration [3]. In the same study, with reference to CT, nodule detection on MRI had 91.7% sensitivity, 100% specificity, 100% positive predictive value, and 100% negative predictive value. The T2W-FSE-P sequence used in that study was disadvantageous due to the long exposure time. It has been stated that the acquisition time takes an average of 3 min, but it can reach 5 min in some patients due to breathing problems. For this reason, the respiratory navigator has been used to prevent motion artefacts that may occur during the examination. Therefore, a respiratory navigator was used herein when obtaining the T2W-FSE-P sequence. In another study conducted by Torkian et al., 8 patients with GGOs, consolidation, reticulation, and reverse halo findings on chest CT were examined. The CT findings were compared with the T2-HASTE and TSE-turbo inversion recovery magnitude (TIRM) sequences. In the statistical analysis performed, the T2W-TSE-TIRM sequence had a higher success in detecting the defined lesions compared to the remaining sequences [24, 25]. Similarly, in the present study, almost complete consistency was found between the chest CT and MRI sequences used in terms of the COVID-19 pneumonia findings.

The first sequences used in the current study were axial and coronal T2W-FSE-P sequences. Lee et al. showed that the T2W-FSE-P sequence had significantly fewer cardiac artefacts and increased boundary sharpness of the tissue interface compared to the conventional T2 TSE sequence [26]. However, in the imaging of the thorax, the T2 FS PROPELLER sequence has been shown to have better quality and less artefacts [27]. Herein, similar to the literature, the MRI sequences had best results compared to CT. The disadvantage of these sequences was their long shooting time. The average shooting time of the axial T2W-FSE-P sequence was 4 min 16 s, and the average shooting time of the coronal T2W-FSE-P sequence was 3 min 10 s. Due to their long acquisition times, a respiratory navigator was used in both sequences.

The second sequence used was axial FIESTA, which is a gradient echo sequence. This sequence was obtained with patients holding their breath. This sequence demonstrates T2/T1-weighing with a high contrast and signal-noise ratio. The most important advantage of this sequence is that it can be obtained with fast sequential acquisition, fat suppression, and overlapping thin sections [26]. According to the shooting parameters used in the current study, the acquisition time of this sequence was 27 s. Compared to CT, the results were almost completely consistent. Thus, axial FIESTA was considered as a useful sequence, especially for patients that experience shortness of breath who cannot hold their breath for a long time.

Another evaluated sequence was axial and coronal T2 SSFSE. This is one of the fast-shooting techniques that allows scanning of the entire lung with patients holding a single breath. In addition, Lee et al. reported that the central k-space oversampling and inherited motion correction properties of this technique resulted in less motion artifacts caused by the heart [26]. According to the parameters used herein, the acquisition time was 1 min for the axial sequence and 45 s for the coronal sequence. The results were very successful.

The last sequence used was the axial T1 LAVA, which is a variant of the T1-weighted fat-saturated gradient-recalled echo. Due to its homogeneous fat suppression property, T1 LAVA is a useful technique, especially for evaluation of the mediastinal and thoracic walls [26]. The acquisition time was 16 s according to the parameters used in this study. Compared to CT, it was very good at identifying pathologies, but produced less successful results compared to the remaining MRI sequences.

In a study by Ekinci at al., they evaluated nodules, consolidation, GGOs, increased patchy density, cavity, bronchodilation, peribronchial thickening, interlobular septal thickening, halo sign, and reverse halo sign in 40 immunocompromized patients with pneumonia [11]. Similar to the preferred sequences in thoracic MRI imaging in the current study, they used T2W balanced fast field echo (B-FFE), T1-weighted turbo spin-echo (TSE), and T2W-TSE sequences in the axial and coronal planes in all of the patients. They compared these MRI sequences with CT. As a result of their study, similar to the findings herein, no significant difference was found between CT and MRI. Among the MRI sequences, the best result was obtained from T2W-TSE. In addition, they noted that the use of a respiratory navigator significantly reduced motion artifacts.

The major difficulties encountered during imaging were artifacts and long imaging times. In the axial sequences, imaging times from the shortest to the longest were determined in T1 LAVA, T2 FIESTA, T2 SSFSE, T2 FS FSE, and finally, T2 FSE-propeller. The imaging time in the coronal sequences was significantly longer than in the T2 FSE-PROPELLER sequence compared to the T2 SSFSE sequence. A respiratory navigator was used due to the long imaging times in the axial and coronal T2W-FSE-P sequences. Other sequences were obtained with the patients holding their breath. According to the clinical condition of the patient, if there was shortness of breath and the patient is not able to hold their breath for a long time, selecting sequences with a shorter imaging time significantly increases the quality of the examination. Intense motion artifacts due to the heart and vascular structures, respiratory artifacts, and susceptibility artifacts due to the low proton density in the lungs make it difficult to evaluate the lung parenchyma with MRI [27]. However, in pulmonary consolidation and GGOs, when the increasing number of protons and signal intensity in infiltration are evaluated together with low signal areas in the adjacent parenchyma, the area with infiltration becomes much more prominent on MRI [28]. In particular, the T2W and contrast-enhanced T1-weighted sequences are very helpful in the diagnosis and differential diagnosis of pneumonia [29].

There were several limitations to this study. The first concerns the assessment being conducted by 2 observers in a single center. Multicenter and observational studies are needed for further and definitive results. The second limitation was that contrast-enhanced sequences were not added to the MRI. It is our belief that when contrast-enhanced sequences are added to the examination, much better results can be obtained.

In conclusion, all 5 sequences evaluated in this study were successful in showing the parenchymal findings of COVID-19. Since the T2W-FSE-P sequence had the best scores in both interobserver agreement and ICC for the image quality score, it can be included in thorax MRI examinations to assist in the diagnosis of COVID-19, especially in pregnant and pediatric patients where radiation exposure should be avoided.

## Figures and Tables

**Table 1:** Clinical and radiological findings of the patients.

Clinical findings	Radiological findings

Mean age: 47.2 ± 24 years	Number of lung lobes affected
	1 lobe in 14 (35.9%) patients
	2 lobes in 10 (25.6%) patients
	3 lobes in 7 (17.9%) patients,
	4 lobes in 5 (12.8%)patients
	5 lobes in 4 (10.3%) patients

Sex	
Male: 22 (52.4%)	Bilateral involvement was affected in 31 (79.5%) patients
Female: 20 (47.6%)	Multifocal involvement was affected in 35 (89.7%) patients

All of the patients had pneumonia clinic	GGOs and consolidation in 25 (64.1%) patients

	Only GGOs in 8 (20.4%) patients
	Only consolidation in 6 (16.5%)

	Air bronchogram in 15 (38.4%) patients
	Crazy paving pattern in 4 (10.2%) patients
	Pleural effusion in 4 (10.2%) patients

**Table 2:** Magnetic resonance sequence parameters.

Sequence	RT/BH	ETL	BW	Matrix	TR	Effective ET	NEX	ST	FOV	Time
Axial Fiesta	BH		83.33	224 × 256	4	1.8	1	5	38	00:27
Axial FS T2 PROPELLER	RT	22	62.5	224 × 256	8.00	66	2	5	38	04:16
Axial T2 FS FSE	BH	15	41.67	224 × 256	2.50	102	1	5	38	01:11
Axial SSFSE	BH		62.50	224 × 256	1.20	120–546	1	5	38	01:00
Axial T1 LAVA	BH		83.33	224 × 256	6.5	2.1	1	5	38	00:19
Coronal FS T2 PROPELLER	RT	22	42.5	224 × 256	1.00	67	1	5	42	03:10
Coronal SSFSE	BH		83.33	224 × 256	1.20	123–476	1	5	42	00:45

FIESTA: fast imaging employing steady-state acquisition, FS: fat-saturated, FSE: fast spin echo, SSFSE: single-shot FSE, LAVA: liver acquisition with volume acquisition, RT: respiratory trigger (triggered by the expiration phase of the respiratory cycle), BH: breath hold, ETL: echo train length, BW: bandwidth, TR: time to repetition, ET: echo time, NEX: number of excitations, ST: slice thickness, FOV: field of view.

**Table 3:** Evaluation of the intersequential interobserver agreement.

Sequence	ICC	95% CI	p-value
T2W-FSE-P	0.913	0.898–0.934	<0.05
SSFSE	0.884	0.864–0.917	<0.05
T2 FSE	0.848	0.821–0.885	<0.05
FIESTA	0.836	0.818–0.851	<0.05
T1 LAVA	0.795	0.764–0.833	<0.05

ICC: Intraclass coefficient, CI: Confidence interval.

**Table 4:** Evaluation of interobserver agreement between the MRI sequences in terms of COVID-19 radiological findings.

Radiological finding	Sequence	ICC	95% CI	p-value
Affected lobes	T2W-FSE-P	1.000	1.000–1.000	<0.05
	SSFSE	0.997	1.000–0.991	<0.05
	T2 FSE	0.982	0.995–0.976	<0.05
	FIESTA	0.912	0.954–0.903	<0.05
	T1LAVA	0.895	0.912–0.881	<0.05
GGO	T2W-FSE-P	0.967	0.981–0.959	<0.05
	SSFSE	0.942	0.954–0.936	<0.05
	T2 FSE	0.927	0.938–0.921	<0.05
	FIESTA	0.883	0.901–0.877	<0.05
	T1 LAVA	0.867	0.883–0.859	<0.05
Consolidation	T2W-FSE-P	0.987	0.991–0.968	<0.05
	SSFSE	0.963	0.985–0.956	<0.05
	T2 FSE	0.934	0.928–0.951	<0.05
	FIESTA	0.895	0.905–0.875	<0.05
	T1LAVA	0.879	0.888–0.857	<0.05
GGO with consolidation	T2W-FSE-P	0.996	0.999–0.989	<0.05
	SSFSE	0.973	0.985–0.966	<0.05
	T2FSE	0.955	0.978–0.951	<0.05
	FIESTA	0.899	0.915–0.887	<0.05
	T1LAVA	0.889	0.898–0.867	<0.05
Crazy paving pattern	T2W-FSE-P	0.996	0.999–0.989	<0.05
	SSFSE	0.973	0.985–0.966	<0.05
	T2FSE	0.955	0.978–0.951	<0.05
	FIESTA	0.899	0.915–0.887	<0.05
	T1LAVA	0.889	0.898–0.867	<0.05

**Table 5:** Interobserver agreement of the lesion-based assessment of the radiological findings.

Radiological finding	Sequence	Kendall’s W	p-value
GGOs	T2W-FSE-P	0.999	<0.05
	SSFSE	0.964	<0.05
	T2FSE	0.958	<0.05
	FIESTA	0.911	<0.05
	T1 LAVA	0.887	<0.05
Consolidation	T2W-FSE-P	0.975	<0.05
	SSFSE	0.957	<0.05
	T2 FSE	0.927	<0.05
	FIESTA	0.915	<0.05
	T1 LAVA	0.881	<0.05
GGOs with consolidation	T2W-FSE-P	0.894	<0.05
	SSFSE	0.885	<0.05
	T2 FSE	0.878	<0.05
	FIESTA	0.815	<0.05
	T1LAVA	0.798	<0.05
Crazy paving pattern	T2W-FSE-P	0.907	<0.05
	SSFSE	0.885	<0.05
	T2 FSE	0.878	<0.05
	FIESTA	0.815	<0.05
	T1 LAVA	0.798	<0.05

GGO: ground-glass opacity.
